# Small-Molecule Inhibition of HIV pre-mRNA Splicing as a Novel Antiretroviral Therapy to Overcome Drug Resistance

**DOI:** 10.1371/journal.ppat.0030159

**Published:** 2007-10-26

**Authors:** Nadia Bakkour, Yea-Lih Lin, Sophie Maire, Lilia Ayadi, Florence Mahuteau-Betzer, Chi Hung Nguyen, Clément Mettling, Pierre Portales, David Grierson, Benoit Chabot, Philippe Jeanteur, Christiane Branlant, Pierre Corbeau, Jamal Tazi

**Affiliations:** 1 Université de Montpellier II, Montpellier, France; 2 Institut de Génétique Moléculaire de Montpellier, Montpellier, France; 3 CNRS, UMR 5535, Montpellier, France; 4 Laboratoire d'Immunologie CHU de Montpellier, Montpellier, France; 5 Institut de Genetique Humaine, Montpellier, France; 6 CNRS, UPR1142, Montpellier, France; 7 Université Henri Poincare-Nancy I, Vandoeuvre-les-Nancy, France; 8 CNRS, UMR 7567, Vandoeuvre-les-Nancy, France; 9 Laboratoire de Pharmaco-chimie, Institut Curie, Orsay, France; 10 CNRS-UMR 176, Orsay, France; 11 Département de Microbiologie et d'Infectiologie, Faculté de Médecine et des Sciences de la Santé, Université de Sherbrooke, Sherbrooke, Québec, Canada; King's College London, United Kingdom

## Abstract

The development of multidrug-resistant viruses compromises antiretroviral therapy efficacy and limits therapeutic options. Therefore, it is an ongoing task to identify new targets for antiretroviral therapy and to develop new drugs. Here, we show that an indole derivative (IDC16) that interferes with exonic splicing enhancer activity of the SR protein splicing factor SF2/ASF suppresses the production of key viral proteins, thereby compromising subsequent synthesis of full-length HIV-1 pre-mRNA and assembly of infectious particles. IDC16 inhibits replication of macrophage- and T cell–tropic laboratory strains, clinical isolates, and strains with high-level resistance to inhibitors of viral protease and reverse transcriptase. Importantly, drug treatment of primary blood cells did not alter splicing profiles of endogenous genes involved in cell cycle transition and apoptosis. Thus, human splicing factors represent novel and promising drug targets for the development of antiretroviral therapies, particularly for the inhibition of multidrug-resistant viruses.

## Author Summary

Over the two decades highly active antiretroviral therapy (HAART) for the treatment of HIV infection has led to a significant decline in morbidity and mortality rates among HIV-infected individuals. HAART uses a combination of molecules that target the virus itself. However, naturally occurring and extensive genetic variation found in the virus allow the emergence of drug-resistant viruses, which rapidly render individuals untreatable. An alternative approach for effective anti-HIV-1 chemotherapy should be targeting cellular factors required for HIV-1 replication. Here, we report the development of a successful therapeutic agent for HIV-1 infection based on inhibition of HIV-1 pre-mRNA splicing, a crucial step of the HIV-1 life cycle that allows production of key viral proteins. The splicing inhibitor IDC16 can efficiently block HIV-1 viral production in primary blood cells infected with different laboratory strains or clinical isolates from patients resistant to anti-HIV multitherapies. These findings may serve as the basis for a new strategy to develop a new class of anti-HIV drugs, the splicing inhibitors, and even of antiviral drugs in general, since any virus needing to splice its RNAs may be targeted.

## Introduction

The increasing prevalence of drug-resistant human immunodeficiency virus type 1 (HIV-1) has highlighted the challenging issue of the optimal treatment of HIV-1-infected patients [1–3]. Current routine drug regimens, typically consisting of various combinations of compounds targeting the viral proteins reverse transcriptase, protease, and gp120, have revolutionized the treatment of HIV/AIDS [3–5]. However, HIV-1 can acquire resistance to all known inhibitors of these targets, and transmission of multidrug-resistant HIV strains is becoming a growing problem [1,2,6–9]. This, as well as other problems such as viral escape mutants [4,10], persistence of viral reservoirs [11–14], poor patient compliance due to complicated regimens [15,16], and toxic side effects [17], have emphasized the need for the development of new drugs with novel mechanisms of action. In addition to virus-specific enzymes, such as reverse transcriptase and protease, several cellular factors are required for replication of HIV-1 [10]. The identification of these critical host cell factors may provide novel cellular targets for the development of compounds that are potentially capable of inhibiting HIV-1, thereby decreasing the burden of viral replication in cases of transmitted multidrug-resistant HIV-1 infection.

To express key viral proteins, HIV-1 uses a combination of several alternative 5′ and 3′ splice sites to generate more than 40 different mRNAs from its full-length genomic pre-mRNA [10]. The choice of these alternative splice sites strongly depends on specific interactions between HIV pre-mRNA sequences and non-spliceosomal nuclear RNA-binding proteins (*trans*-acting factors). These *trans*-acting factors can be classified as SR proteins (serine-arginine-rich proteins) [18–21] and hnRNPs (heterogenous nuclear ribonucleoproteins) [22–25]. Binding of the SR proteins to exonic splicing enhancers (ESEs) downstream of the Tat-, Rev-, Vpr-, Env-, and Nef-specific 3′ splice sites promotes exon definition by recruiting constitutive factors and preventing the action of nearby splicing silencers [26]. Therefore, members of the SR protein family are thought to play a major role in the regulation of HIV-1 pre-mRNA splicing.

By screening a large collection of chemical compounds, our laboratory has discovered several benzopyridoindole and pyridocarbazole derivatives that selectively inhibit the ESE-dependent splicing activity of individual SR proteins [27–29]. Selective binding of these compounds to specific SR proteins prevents spliceosome assembly and splicing in an in vitro system. Given the fact that these proteins play an important role in regulating HIV-1 pre-mRNA splicing, we surmised that specific inhibition of SR proteins by benzopyridoindole and pyridocarbazole derivatives could block HIV-1 replication.

## Results

**Figure 1:**
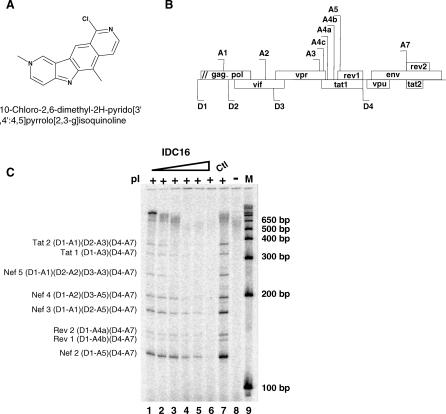
Selective Inhibition of HIV-1 RNA Splicing *Ex Vivo* by IDC16 (A) Drawing and formula of IDC16 compound. (B) Schematic representation of HIV-1 genome. The 5′ splice sites (D1–D4) and 3′ splice sites (A1–A7) are indicated. The various open reading frames are boxed. (C) HeLa cells transfected with the pΔPSP construct were either untreated (lane 7) or treated with 0.05 μM, 0.1 μM, 0.5 μM, 1 μM, 2.5 μM, or 5 μM of compound IDC16 (lanes 1–6, respectively). Multiply spliced products of HIV-1 RNA were amplified by RT-PCR using the oligonucleotide primers BSS and SJ4.7A. The PCR products were analyzed by polyacrylamide gel electrophoresis after normalization with *GAPDH* (see Materials and Methods). Nomenclature of the RT-PCR products on the left of the panel is according to [30]. Size markers (in bp) are shown on the right of the panel (lane 9). RT-PCR from untransfected HeLa cells (lane 8).

Among 220 indole derivatives that were screened for selective inhibition of ESE-dependent splicing events, one selected drug (IDC16) demonstrated a strong inhibitory effect on different substrates harboring SF2/ASF high-affinity binding site (Figure 1A). IDC16 had also no adverse effect on cell growth and viability of different cell lines, including primary cell lines (see below). Given that SF2/ASF plays a major role in HIV-1 pre-mRNA splicing, we decided in the present study to test the effect of IDC16 on HIV replication as well as on specific splicing events of the HIV-1 pre-mRNA. The efficiency of the drug was first assessed by using the pΔPSP plasmid containing the HIV-1 proviral genome deleted between nucleotides 1511 and 4550 (Figure 1B), which recapitulates all splicing events of HIV-1 pre-mRNA in transfected HeLa cells [30]. The mRNAs produced by splicing were then analyzed by reverse transcriptase (RT)-PCR using forward and reverse primers that amplify several splicing isoforms encoding the viral proteins Nef, Rev, and Tat. Compared to untreated cells, the synthesis of all splicing products was less efficient (compare lane 7 and lanes 1–6). The effect of IDC16 was dose dependent, especially for the synthesis of larger splicing isoforms, with a complete block at 2.5 μM. At this concentration of the drug neither global nor HIV-1 RNA synthesis were affected (unpublished data, see below), implying that IDC16 has a specific action on HIV-1 pre-mRNA splicing.

**Figure 2:**
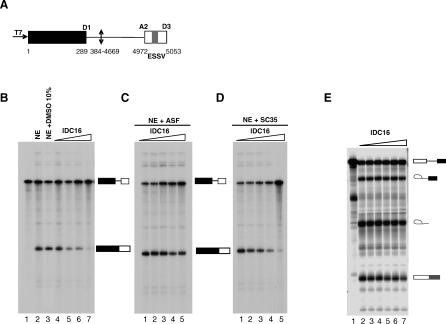
In Vitro Inhibition of HIV1-D1-A2 pre-mRNA Splicing by IDC16 and Complementation with SR Proteins SC35 and SF2/ASF (A) Schematic representation of HIV1-D1-A2 transcript is shown. Numbering of the HIV-1/BRU RNA sequences is according to Ratner et al. [49]. (B) Polyacrylamide gel electrophoresis of the in vitro splicing products of HIV1-D1-A2 transcript. Uniformly labeled RNA was prepared as described under Materials and Methods and was incubated alone (lane 1) in a HeLa cell nuclear extract under splicing conditions in the absence (lane 2), in the presence of 10% DMSO (lane 3), or 5 μM, 10 μM, 20 μM, and 40 μM of compound IDC16 (lanes 4–7, respectively). (C) The same reactions as in (B) lanes 3–7 were complemented with 0.21 μM of recombinant SF2/ASF (lanes 1–5, respectively). (D) The same reactions as in (B) lanes 3–7 were complemented with 0.19 μM of recombinant SC35 (lanes 1–5, respectively). (E) Splicing products of the Minx transcript analyzed by polyacrylamide gel electrophoresis. Uniformly labeled RNA was incubated alone (lane 1), in HeLa nuclear extract in the absence (lane 2) or presence of 5 μM, 10 μM, 20 μM, 30 μM, or 50 μM of compound IDC16 (lanes 3–7), respectively.

The dose-dependent profile of splicing inhibition indicated that IDC16 inhibits the use of several weak 3′ splice sites whose utilization is required for the production of key viral regulatory proteins. Utilization of these 3′ splice sites is known to critically depend upon the binding of SR proteins [31]. To examine the specificity of IDC16, we tested the effect of this drug in an in vitro splicing assay using a pre-mRNA substrate (HIV1-D1-A2) harboring the D1 and A2 HIV donor and acceptor splice sites, respectively (see Figure 2A, [32]). Splicing of this substrate is activated by the SR protein ASF/SF2, both in vitro and *ex vivo*, and therefore it constitutes an ideal target for IDC16, which inhibits most of SF2/ASF ESE-dependent splicing tested ([26] and unpublished data). Treatment with increasing concentrations of IDC16 inhibits splicing of HIV1-D1-A2 in a dose-dependent manner (Figure 2B). Complementation of the extract with recombinant SR protein ASF/SF2 strongly limited the IDC16 splicing inhibition (Figure 2C), whereas the addition of similar amounts of another SR protein, SC35, fails to do so (Figure 2D), demonstrating that IDC16 specifically impedes SF2/ASF-dependent splicing. Consistently, concentration up to 50 μM of IDC16 did not alter splicing of synthetic mRNA precursors derived from the adenovirus major late-transcription unit (Minx, Figure 2E), which is a single intron pre-mRNA not requiring ESE sequences in the second exon for efficient splicing.

**Figure 3:**
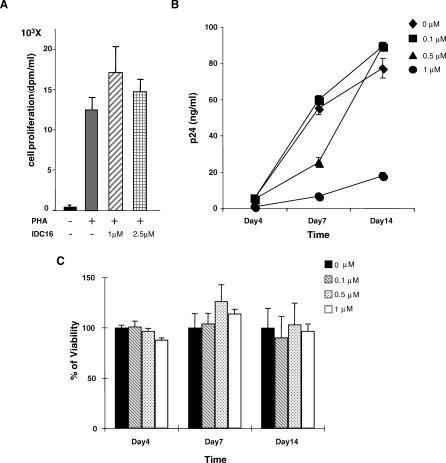
Inhibition of HIV-1 Production in PBMC-Infected Cells by IDC16 (A) PBMCs from healthy donors were cultured in the presence of labeled ^3^H-thymidine and incorporated radioactivity was measured after 72 h stimulation with phytohemagglutinin A (PHA) in the absence (-) or presence of 1 μM or 2.5 μM of IDC16. (B) 100 TCID_50_ of Ada-M was used to infect triplicate of 10^6^ activated PBMCs (stimulated for 2 d with PHA and IL2) in the absence or presence of 0.1 μM, 0.5 μM, or 1 μM of IDC16. Supernatant was harvested at the indicated days and viral capsid protein p24 antigen was quantitated using standard ELISA protocol. (C) Concurrently, cell viability was measured by trypan blue exclusion and indicated as percentage as compared with untreated cells.

Given the key role played by ASF/SF2 in the activation of several HIV-1 acceptor sites, it is expected that the treatment of infected cells with IDC16 might block HIV-1 replication. Initial experiments were designed to determine the concentration of IDC16 with minimal side effects on cell viability and cell cycle progression. Treatment of stimulated peripheral blood mononuclear cells (PBMCs) with 1 or 2.5 μM IDC16 next showed no effect on cell proliferation, as measured by tritiated thymidine incorporation into cellular DNA (Figure 3A). Since no adverse cellular effect was observed up to the 2.5 μM IDC16 concentration range, we then asked whether IDC16 could block HIV-1 replication in this potential therapeutic window. Stimulated PBMCs were infected with either NL4.3 (unpublished data) or Ada-M R5 (Figure 3B) HIV-1 strains and cultured for 14 d in the presence or absence of 0.1 μM, 0.5 μM, or 1 μM IDC16. At the indicated days, virus replication was determined by p24 antigen enzyme-linked immunosorbent assay (ELISA). Replication of both NL4.3 (unpublished data) and Ada-M R5 strains (Figure 3B) is very efficiently blocked in these primary cells following treatment with 1 μM of IDC16. Meanwhile, cell viability was not affected by the drug throughout the assay as shown in Figure 3C.

**Figure 4:**
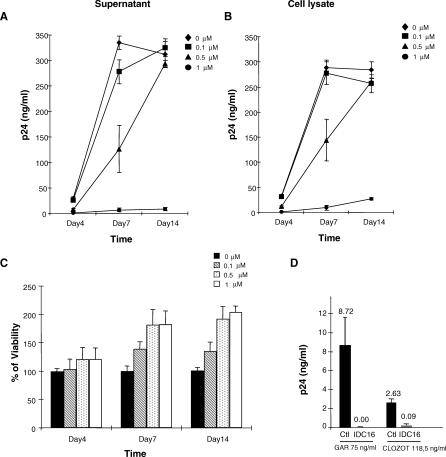
Inhibition of HIV-1 Production in Macrophage-Infected Cells by IDC16 Macrophages were infected with 100 TCID_50_ of Ada-M for 18 h in the absence or presence of 0.1 μM, 0.5 μM, or 1 μM concentrations of IDC16 and then washed intensively with PBS. (A and B) The culture medium and cells were collected at days 4, 7, and 14, and extracellular (A) or intracellular (B) viral production was measured by the quantification of viral capsid protein p24 using ELISA. (C) Concurrently, cell viability was measured by trypan blue exclusion and indicated as percentage as compared with untreated cells. (D) Activated PBMCs from healthy donors were infected with two HIV-1 clinical isolates (GAR and CLO) in the absence (Ctl) or presence of 1 μM of IDC16 (1DC16). Supernatants were harvested after 14 d of infection days, and viral capsid protein p24 antigen was quantitated using standard ELISA protocol.

To generalize the effect of IDC16 on HIV-1 replication in other primary cells, the same protocol was repeated using infected macrophages, which act as viral reservoirs. Cells were treated with 0.1 μM, 0.5 μM, or 1 μM of IDC16 and p24 antigen levels were monitored both in culture supernatant (Figure 4A) and in cell lysates (Figure 4B). Again, IDC16 blocked virus replication efficiently and in a dose-dependent manner, reaching inhibition levels up to 90% in primary macrophages at 1 μM. However, cell viability was not decreased under IDC16 treatment (Figure 4C). It is important to note that macrophages survival was rather increased in the presence of IDC16 at day 7 and day 14 post-infection (Figure 4C), suggesting that IDC16 may protect these cells from deleterious effects induced by viral infection.

The previous experiments were all performed with primary human cells infected with either macrophage-tropic (R5) HIV-1, Ada-M, or T-cell-line-tropic (X4), NL4–3 HIV-1 laboratory strains, suggesting that IDC16 could be effective on a variety of viral strains detected in vivo. We next used an in vitro system that may be more relevant to the clinical situation, since it involves infecting primary cells with HIV-1 isolates from patients resistant to conventional antiretroviral therapies. We chose five extreme cases in which viruses harbored mutations in different regions of the viral genome, including those encoding the reverse transcriptase and protease domains. Using two strains that previously demonstrated robust resistance to different therapeutic agents in vitro, we show that IDC16 also very efficiently inhibits the replication of these clinical isolates, since no viral particles were detected 14 d post-infection (Figure 4D).

**Figure 5:**
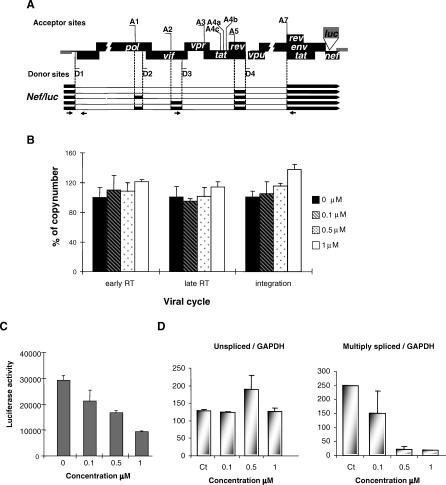
Identification of the Step in HIV Life Cycle Inhibited by IDC16 HOS-CD4^+^-CCR5^+^ cells were exposed to various concentrations of IDC16 and infected with Δenv-defective virions containing the *luciferase* gene (A). The efficiency of the reverse transcription and of the integration was evaluated by quantifying early and late reverse transcriptase products and the number of copies of integrated HIV DNA, respectively (B). Forty eight hours later, intracellular luciferase activity was measured as a marker of spliced viral RNA expression (C). The same infected cells treated with 0.1 μM, 0.5 μM, or 1 μM of IDC16 were used to examine the relative amount of unspliced precursor and multiply spliced HIV-1 RNA. Real-time RT-PCR was used to rigorously quantify the changes in unspliced (D, left panel) and multiply spliced (D, right panel) HIV-1 RNA levels after IDC16 treatment. The values are the average of two independent experiments and the level of expression is normalized with that of the internal control gene (*GAPDH*). The error bars indicate standard deviations. Arrows in (A) indicate the position of primers to amplify unspliced precursors (La 8.1 and La 9) and multiply spliced (P659 and P413MOD) HIV RNA species according to Brussel and Sonigo [48].

In order to provide further evidence that the anti-HIV activity we observed with IDC16 is actually the consequence of its inhibitory effect on viral RNA splicing, we examined various steps of the viral cycle in cells treated with the drug and submitted to one-round infection. For this purpose, we exposed HOS-CD4^+^-CCR5^+^ cells to defective virions obtained by cotransfecting 293T cells with a plasmid encoding the R5 envelope of the AD8 strain and another plasmid containing the entire HIV-1 genome mutated in the *envelope* gene and harboring a *luciferase* marker gene fused to *nef* (Figure 5A, [33]). The amounts of luciferase activity in cells infected with these virions reflect both the number of integrated proviruses and expression of multiply spliced species encoding nef/luc (Figure 5A). Two days post-infection, cells were lysed and the amount of early reverse transcriptase products (strong-stop fragment), of late reverse transcriptase products (LTR-gag fragment), and of integrated proviral DNA (Alu-LTR fragment) was measured by quantitative PCR. At all concentrations tested, the drug had no effect on early reverse transcription, late reverse transcription, or integration (Figure 5B). In contrast, Figure 5C shows the dose-dependent inhibition by IDC16 of luciferase activity in HOS-CD4^+^-CCR5^+^-infected cells. Of note, the inhibitory effect could be smaller in this one-round infection assay than in the previous assays, where several rounds of infection were carried out.

To evaluate the effect of IDC16 on the expression of unspliced and multiply spliced HIV-1 RNA species in HOS-CD4^+^-CCR5^+^-infected cells, we used real-time RT-PCR to analyze the changes in mRNA levels. The results (Figure 5D, left panel) show that treatment of infected cells with 0.1 μM and 1 μM concentrations of IDC16 did not change the level of unspliced mRNA species using *GAPDH* mRNA as a reference. A significant increase of these species was, however, observed when cells were treated with 0.5 μM of IDC16. In sharp contrast, IDC16 treatment induced a dose-dependent decrease of multiply spliced species (Figure 5D, right panel), confirming that the drug actually inhibited HIV RNA splicing. Also, consistent with the current view that the production of full-length HIV-1 RNA requires factors encoded by multiply spliced species, like Rev and tat, the large decrease of multiply spliced species induced by IDC16 treatment has not been compensated by a similar increase of unspliced HIV-1 RNA.

**Figure 6:**
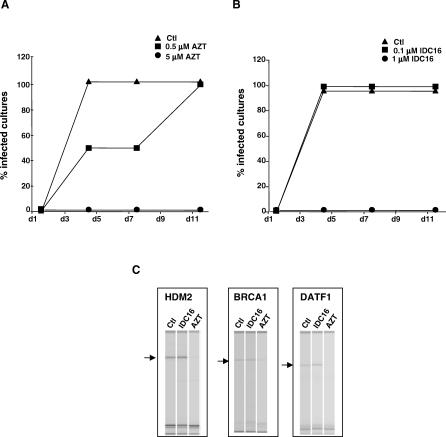
Comparison of HIV-1 Inhibition in MT2 Cells Treated with AZT or IDC16 and Analysis of Splicing Profiles of Apoptotic Genes in PBMC-Treated Cells (A and B) MT2 cells cultured in a 96-well plate were infected with pNL4.3 at 100 TCID_50_ in the absence or presence of IDC16 (A) or AZT (B) for 18 h. Cells were then washed and changed to fresh medium with/without IDC16 or AZT. Half of the culture medium was refreshed each 2 or 4 d in the presence of drugs. The formation of syncytia was scored at the indicated time points. (C) Four ug of total RNA from triplicate of PBMCs untreated (lane Ctl) or treated with IDC16 (lane IDC16) or AZT (lane AZT), was reverse transcribed with Omniscript reverse transcriptase (QIAGEN) using random hexamers and oligo dT and. The mixture was aliquoted in a 96-well plate and subjected to PCR amplification using 0.375U/15 μl of hotStarTaq DNA Polymerase with specific primers (0.3–0.6 μM) using the buffer provided by the manufacturer (QIAGEN). The PCR reaction was carried out in a GeneAmp 9700 PCR system. Following an incubation of 15 min at 95 °C, and 35 cycles of 30 s at 94 °C, 30 s at 55 °C, and 1 min at 72 °C, the reaction was ended with an extension step of 10 min at 72 °C. PCR products were fractionated on a LabChip HT DNA assay station (Caliper) for quantitation and sizing. The full data can be accessed through http://www.lgfus.ca/Tazi/, username = Tazi, password = sc35. Three examples of genes altered by AZT treatment (HDM2), (BRCA1), and (DATF1) are shown.

All drugs that inhibit viral production also inhibit cell–cell fusions; we therefore assessed the capacity of IDC16 to inhibit the fusion of HIV-1-infected cells compared to azidothymidine (AZT, 3′-azido-3′-deoxythymidine, zidovudine). AZT is the first nucleoside reverse transcriptase inhibitor approved for HIV-1 therapy [34]. Its antiretroviral activity is likely to involve at least two steps: incorporation into viral DNA and inhibition of the viral reverse transcriptase. While incorporation of the drug into host nuclear and mitochondrial DNA may be largely responsible for dose-limiting toxicities, AZT remains a potent and frequently prescribed antiretroviral therapy for HIV-positive individuals. We therefore subjected both AZT and ICD16 to HIV-1 inhibition tests in infected MT2 cultures to compare their relative antiretroviral effects. MT2 cells cultured in a 96-well plate were infected with pNL4.3 at 100 TCID_50_ in the absence or the presence of IDC16 or AZT for 18 h. Cells were then washed and changed to fresh medium with or without IDC16 or AZT. Half of the culture medium was refreshed each 2 or 4 d in the presence of drugs. The formation of syncytia was scored at the indicated time points. Results illustrated in Figure 6A and 6B show that viral infectivity is completely abolished using five times less IDC16 as opposed to AZT. In fact, at a concentration of 1 μM, IDC16 is able to reduce the percentage of infected cultures to nearly nil, whereas 5 μM of AZT were needed in order to observe the same effects, indicating that effective inhibitory concentration within cellular systems of compound IDC16 is in the range of that of AZT.

Since we are promoting SR proteins as an attractive intracellular target of anti-HIV therapies, it was necessary to demonstrate that the antiviral effect of compound IDC16 by inhibiting SR proteins did not have a global effect on the splicing of endogenous genes. To address this question and to determine the general impact of the compounds on alternative splicing, we selected a set of 96 alternative splicing units covering a variety of human apoptotic genes. RT-PCR analysis on RNA extracted from triplicate PBMCs treated or not with compound IDC16 or AZT revealed the existence of multiple amplification products for 81 of them. The relative abundance of these different products was not affected by compound IDC16, suggesting no large impact on alternative splicing (the full data can be accessed through http://www.lgfus.ca/Tazi/, username = Tazi, password = sc35). Among this set, however, three displayed reduced levels in the presence of AZT (Figure 6C): breast and ovarian cancer susceptibility (BRCA1, see Table 1), human homolog of double minute 2 (HDM2, see Table 1), and death inducer-obliterator 1 (DATF1, see Table 1). Thus, AZT, an antiretroviral agent with proven clinical benefit for the treatment of HIV/AIDS, might have a detrimental effect on cell survival since the genes whose expression is altered in AZT-treated cells play a critical role in maintaining genome integrity [35–37]. These results are unexpected because AZT specifically targets HIV-1 reverse transcriptase, and therefore it is not supposed to alter the expression of endogenous genes. Hence, through analysis of a small fraction of selected genes, it was possible to show that AZT but not IDC16 alters host cell gene expression.

**Table 1:**
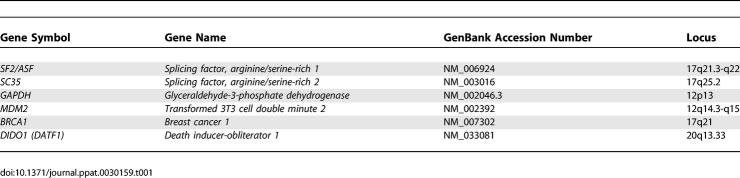
List of Genes Mentioned in This Manuscript

## Discussion

Taken together, our data indicate that IDC16 can efficiently block HIV-1 viral production in PBMCs or macrophages infected with different laboratory strains or clinical isolates from patients resistant to anti-HIV multitherapies. As IDC16 acts on a cellular factor, the risk of resistance development should be reduced. Indeed, to escape from IDC16 inhibition, a strain should have to mutate its ESE into a new one, requiring another SR protein than SF2/AF. IDC16 could thus represent the first member of a new class of anti-HIV drugs, the splicing inhibitors, and even of antiviral drugs in general, since any virus needing to splice its RNAs may be targeted.

To help develop this novel class of anti-viral inhibitors, several aspects of SR functions need now to be considered. First, we will have to address the phosphorylation status and/or specific localization of SR proteins in response to drug treatment. Indeed, indole derivatives have been shown to bind directly to the RS domain of SR proteins and thereby impede its phosphorylation, at least in vitro, by SR kinases [27–29]. Since phosphorylation affects both splicing activity and subcellular trafficking of SR proteins [38], treatment of cells with IDC16 may modulate these two processes and act synergistically to modify HIV-1 RNA splicing and/or export. Second and most important, we will have to determine whether IDC16 will affect additional functions that SR proteins have during gene expression, like mRNA export [39–42], mRNA stability [43], stimulation of mRNA translation [44], or maintenance of genomic stability [45]. Our current studies are now aimed at confirming in vivo lack of deleterious side effects of this molecule by comparing the profiles of SF2/ASF depletion and inhibition by IDC16. The finding that IDC16, unlike SF2/ASF depletion, could exhibit a low cellular toxicity already indicates that this molecule is selective for some functions of SF2/ASF that are shared by other SR proteins. Along this line, it is noteworthy that most of the drugs selected in our initial in vitro splicing inhibition screen were not previously considered as good candidates for use in cancer therapy because of their low cytotoxicity [29]. Furthermore, IDC16 has more impact on HIV-1 alternative splicing than that of endogenous gene (present study). While IDC16-mediated splicing modulation has been tested only for few genes, a likely explanation for this difference of splicing inhibition behavior could be that the viral RNA has to escape the splicing machinery during later stages of infection to produce viral particles containing full-length unspliced pre-mRNA, whereas most cellular genes have constitutive exons that contain redundant binding sites for SR proteins. A robust and comprehensive exon microarray that can detect with high accuracy alteration of splicing events is needed to consolidate this hypothesis. Available tools are still at a validation stage and are not sensitive enough to monitor the transcripts variation generated by splicing at large-scale to cover the expression of whole human genome.

Our results are consistent with the notion that cellular targets, like the SR proteins, can be used as potent targets to overcome drug resistance resulting from highly active antiretroviral therapy. Moreover, genes encoding cellular proteins do not mutate under physiological conditions, and one could expect that HIV-1 resistance to IDC16 would occur far less frequently than resistance to a conventional drug targeting viral proteins. More importantly, enhancer sequences bound by SR proteins are essential for efficient splicing, a prerequisite step to viral replication. Viruses that harbor mutations in these enhancers due to reverse transcriptase errors would have very little or no chance of survival. Similarly, mutations that improve the binding of SR proteins will also be detrimental for viral replication, as they will impede the production of full-length HIV-1 mRNA.

## Materials and Methods

### Chemical library.

The Institut Curie–CNRS chemical library contains 6,720 molecules kept in 96 well microplates at a concentration of 10 mg/ml in dimethyl sulfoxide (DMSO). Extemporaneous dilutions were made with 10% DMSO. Microplates were kept at −20 °C.

### Recombinant proteins and in vitro and *ex vivo* splicing assays.

Recombinant SF2/ASF and SC35 were produced and purified from baculovirus-infected Sf9 cells as previously reported [46].

The HIV1-D1-A2 plasmid has already been described [32]. In vitro transcription to obtain radiolabeled transcripts and splicing reactions were performed under standard conditions for 1 h as described [32] in the presence of the indicated concentration of IDC16. Splicing products were analyzed by electrophoresis on denaturing 7% polyacrylamide gels and revealed by autoradiography.

HeLa cells (5 × 10^5^ cells) were grown in RPMI 1640 (GIBCO BRL), supplemented with 10% fetal calf serum (FCS) on 3-cm diameter dishes (Nunc) to 70%–80% confluence. Transient transfections with splicing reporter constructs (1 μg) were performed with the LipofectAMINE Plus reagent (Invitrogen) according to the manufacturer's instructions.

Total cellular RNA was isolated from transfected HeLa cells 48 h post-transfection, and 3 μg of RNA was reversed transcribed and PCR amplified with forward primer BSS (5′-GGCTTGCTGAAGCGCGCACGGCAAGAGG-3′; nt 700–727) and reverse primer SJ4.7A, which spans sites D4 and A7 (5′-TTGGGAGGTGGGTTGCTTTGATAGAG-3′; nt 8369–8381 and 6032–6044) [30]. To normalize the signals, *GAPDH* was used as an internal control of the PCR reactions as described [27]. Amplification products were radiolabeled by performing a single round of PCR with the addition of 10 μCi of [α-^32^P]dCTP, and the products were analyzed by electrophoresis on 6% polyacrylamide 8 M urea gel as described [30].

### Preparation and infection of primary human macrophages and T cells.

Human PBMCs from healthy donors were isolated by Ficoll-Paque density centrifugation. They were then cultured in RPMI plus 1% heat-inactivated human serum AB at a concentration of 2.5 × 10^6^ cells/ml and then incubated at 37 °C, 5% CO_2_ for 1 h in 24-well plates. After removing the non-adherent cells, the adherent cells were kept in complete RPMI with 10% FCS at 37 °C for another 2 h. Ten U/ml GM-CSF (Roche) was added to the culture medium and incubated for 4 d before viral infection.

For HIV-1 infection, human PBMCs and macrophages were infected with 100 TCID_50_ of Ada-M (R5 strain) for 18 h in the absence or presence of various concentrations of IDC16 and then washed intensively with PBS. The culture medium and cells were collected at day 4, 7, and 14. Viral production was measured by the quantification of viral capsid protein p24 using ELISA (Beckman Coulter). Cell viability was quantified by trypan blue exclusion.

MT2 cells cultured in quadriplicate in a 96-well plate were infected with NL4–3 at 100 TCID_50_ in the absence or presence of IDC16 or AZT for 18 h. Cells were then washed and changed to fresh medium with/without IDC16 or AZT. Half of the culture medium was refreshed each 2 or 4 d in the presence of drugs. The formation of syncytia was scored at the indicated time points.

### Pseudotyped virion production and single-round infection assays.

The plasmid pNL4.3-env^−^-Luc^+^ harboring a *luciferase* gene was co-transfected with the envelop plasmid pCMV-Ad8-Env (NIH AIDS Research and Reference Reagent Program) into human embryonic kidney cells-293T to produce R5-pseudotyped virions. Human osteosarcoma (HOS)-CD4^+^-CCR5^+^ cells (from the NIH AIDS Research and Reference Reagent Program) were treated with or without drug and infected for 18 h, washed three times with PBS, and kept in fresh RPMI-1640 containing 10% FCS with or without drug. Luciferase activity was monitored 48 h post-infection using a luciferase assay kit and a luminometer according the manufacturer's instructions (Promega).

At 48 h post-infection with Δenv-defective viruses, HOS-CD4^+^-CCR5^+^ cells were resuspended in lysis buffer (10 mM Tris [pH 8.0]; 0.5 mM EDTA; 0.0001% SDS; 0.001% Triton; 100 μg/ml Proteinase K), incubated 3 h at 50 °C and 10 min at 95 °C. For strong-stop and late reverse transcripts, DNA was amplified with the appropriate primers at 70 °C in a LightCycler (Roche) with SYBR Green following the manufacturer's recommendation. Viral DNA was normalized by cellular genomic CCR5. Integration was measured using Alu-LTR-based real-time nested PCR procedure according to Brussel et al. [47], with the following modifications: the first amplification with primers L-M667 only (control) or with Alu1 and Alu2 (integrated) had an annealing temperature of 65 °C. To reduce unspecific background, 2 μl of the first amplification was digested with 20 U of Exonuclease I (New England Biolabs) in 20 μl for 2 h at 37 °C. The nuclease was heat inactivated at 80 °C for 20 min. Two μl of the digestion was then amplified with SYBR Green at 65 °C with primers Lambda T and AA55M. Primers sequence: Strong-stop: (1) agcctgggagctctctggcta and (2) ccagagtcacacaacagacgg; Late: (1) and (3) cgcttcagcaagccgagtcct; CCR5 gene: (4) gtgaagcaaatcgcagcccgc and (5) gcagcatagtgagcccagaag.

To quantify the unspliced and multiply spliced HIV-1 RNA, 5 μg of total RNA was extracted and reverse transcribed using first strand synthesis reverse transcription kit (Invitrogen) (5 μg of RNA were used without Reverse Transcription Enzyme as a negative control). Real-time PCR (Bio-Rad) was used to amplify both unspliced HIV-1 RNA using primers La8.1 (CTGAAGCGCGCACGGCAA) and L9 (GACGCTCTCGCACCCATCTC) and multiply spliced HIV-1 RNA using primers p659 (GACTCATCAAGTTTCTCTATCAAA) and p413MOD (AGTCTCTCAAGCGGTGGT) as described previously [48]. The amount of RNA was normalized to GAPDH mRNA.

### Alternative splicing profiling.

Four ug of total RNA was reverse transcribed with Omniscript reverse transcriptase (QIAGEN) using random hexamers and oligo dT. The mixture was aliquoted in a 96-well plate and subjected to PCR amplification using 0.375 U/15 μl of hotStarTaq DNA Polymerase with specific primers (0.3–0.6 μM) using the buffer provided by the manufacturer (QIAGEN). The PCR reaction was carried out in a GeneAmp 9700 PCR system. Following an incubation of 15 min at 95 °C, and 35 cycles of 30 s at 94 °C, 30 s at 55 °C, and 1 min at 72 °C, the reaction was ended with an extension step of 10 min at 72 °C. PCR products were fractionated on a LabChip HT DNA assay station (Caliper) for quantitation and sizing.

## Abbreviations

AZT: azidothymidine (3′-azido-3′-deoxythymidine, zidovudine)
ELISA: enzyme-linked immunosorbent assay
ESE: exonic splicing enhancer
HIV-1: human immunodeficiency virus type 1
PBMC: peripheral blood mononuclear cell
SR: serine-arginine-rich
